# Medical criteria of pathologicity and their role in scientific psychiatry—comments on the articles of Henrik Walter and Marco Stier

**DOI:** 10.3389/fpsyg.2014.00128

**Published:** 2014-02-19

**Authors:** Peter Hucklenbroich

**Affiliations:** Institute for Medical Ethics, History and Philosophy of Medicine, University of MuensterMuenster, Germany

**Keywords:** pathologicity, disease entities, disease criteria, naturalism-normativism-controversy

The articles of Walter (2013) and Stier (2013) refer to some central problems of philosophy regarding the concept of disease. Among them are the questions whether psychiatric diseases exist at all, how they are to be distinguished from mere problems of everyday life, which underlying theoretical concept of disease is assumed, and what kind of normativity—if any—is associated with their definition. In this commentary, I am going to commemorate the way medicine in general identifies and defines diseases and kinds of diseases, and the role theoretical concepts like *pathologicity* and *disease entity* play in medical epistemology and concept formation. The fundamental ideas and principles of modern medical pathology (German: *Krankheitslehre*) are the following ones:

1. Diseases are circumscribed, partial processes and conditions of the life process of human individuals as a whole. They are not a necessary part of life, i.e., a life without any disease or pathological condition is theoretically possible. Processes and conditions that are necessary, inevitable parts of life (e.g., developmental stages like childhood) are, in themselves, never pathological conditions (but of course they may be *pathologically altered*).
2. *Diseases* are generally distinguished from healthy, normal conditions insofar as they meet at least one *disease criterion* (or *criterion of pathologicity*).
3. There are five primary criteria of pathologicity:
  - Shortening of lifetime expectancy, or immediate lethality
  - Pain, and other specific somatic or vegetative complaints
  - Infertility, i.e., inability of biological reproduction
  - Inability or impairment of living together in human symbiotic communities
  - Non-universal *disposition* of the organism to develop a condition that is pathological according to one or more of these criteria (this clause covers also conditions that are usually called risk/risk factor, disability, impairment, or handicap).
4. Diseases originate from circumscribed, first or primal causes (or complexes of first causes) that interact from the outside (i.e., from the environment or the parental generation) with the life process of the individual.
5. All partial processes of a disease are *causally* connected. Particularly and retrospectively, they would have occurred even without or against the individual's conscious, intentional volition.
6. In medical theory, the entirety of possible pathological processes forms a huge but definite, manageable system of pathological conditions and causal pathomechanisms that represents the subject matter of General and Special Pathology (including Pathophysiology and Pathobiochemistry).
7. In medical theory, the entirety of diseases may be systematized and classified according to their causal structure and clinical features, and is classified into species (kinds) of diseases that are called *disease entities* and arranged taxonomically by Nosology.

These seven principles represent core theoretical ideas of modern medicine. Around these core ideas the entire existing medical theory of diseases has developed in history and can be reconstructed by philosophy^1^.

In the present context of theoretical pathology several remarks may deserve particular attention:

1. Criteria of pathologicity do not express valuations or normative assessments, but form criteria for the use of *discriminating* and *classifying* conditions of life and living organisms. Particularly, this applies to conditions of pain and bodily or vegetative complaints and discomfort: Indeed, these kinds of sensation are experienced as negative, unpleasant or even unbearable and insufferable events, but their “negativity” is not the result of a free, intentionally eligible evaluation, or of a socio-culturally established norm or convention, but is *determined by nature* (viz. the nature of the human organism). It has developed and been selected in the course of natural evolution and phylogenesis of the species *homo sapiens*. This fact is not contradicted by a different fact: that pain and discomfort of this kind are, *additionally*, in most—but, interestingly, not in all—cases of occurrence *evaluated* negative by the affected individual, and are subsumed by the socio-cultural environment under the *norm* of being in need of treatment. These evaluations and normative assessments form an additional over-determination of the natural, physiologically determined sensations, and they may, in distinction to the natural sensation, be denied (e.g., in asceticism) or even inverted (e.g., in self-punishment or self-mutilation).
2. The above principle 2 postulates a strict dependence of pathologicity on criteria 1-5. But this principle is overwritten by a theoretically more elaborate account of pathology and nosology: There are single instances of disease entities that do not meet any primary criterion of pathologicity. Nevertheless, they are classified as diseases, because they meet the *defining criteria of the respective disease entity*. These defining criteria may be, e.g., definite changes in cell structure, or in composition of blood, that can be ascertained by pure lab findings. In such cases, theoretical classification overrides and overwrites phenomenological classification. This procedure that is common in medicine shows that, in principle, identification of diseases and of pathologicity has “emancipated” from the pure phenomenological level and the level of pure clinical symptoms, and will give priority to the theoretical system in ambiguous cases.

Recent philosophical debates on the concept of disease have been paralyzed by the controversy between naturalism and normativism. Naturalism attributes this concept to differences given in nature, while normativism ascribes these differences to individual or socio-cultural valuations and preferences^2^. The reconstruction of medical theory of disease sketched above shows this dichotomy and antagonism to be misguided: In the first instance, the conditions and circumstances addressed in the criteria of pathologicity are naturally given possibilities resulting from the structure and organization of human organism and its embedment into its natural environment:

- mortality and lethal vulnerability are determined by the very structure of living organisms
- negative sensations like pain, nausea, tussive irritation, pruritus (itching) etc. belong to protective mechanisms that are universal features of human organisms
- ability of biological reproduction is naturally (biologically) represented in the male and female reproductive systems consisting of specialized organs, hormones, functions, and mental affections
- living together in (human) symbiotic communities is prerequisite for survival of every human individual, from birth and maternal care to life-long cooperation and mutual assistance.

The last feature, of living together, may be realized in very different, culturally determined and historically changing ways. This openness to variance shapes deeply the form of symbiosis but does not suspend the universal necessity of symbiosis at all. Thus, criteria of pathologicity possess a foundation in nature and are not constituted by valuations or social norms, but by the natural, bio-psycho-social life-form of homo sapiens. However, defending this position does not imply that one is bound to deny, ignore, or underestimate the cultural and historical embedding and variability concerning forms of thinking and social life dealing with phenomena of disease. On the contrary: Cultural values and traditions as well as individual convictions and preferences are decisive regarding the way disease phenomena are interpreted, evaluated, and regulated by norms and institutions. Help and assistance for ill persons, emergence of the professional role of physician, and the development of public health systems are intentional and socio-cultural *reactions* to the natural phenomenon of disease. In this sense and in the final phenomenon, disease—also, mental disease—is *simultaneously* determined by natural and by socio-cultural factors. One-sided naturalism as well as one-sided normativism both are misleading.

The article of Henrik Walter provides an impressive account of the development and actual status of biological psychiatry. Only the status of the concept of disease remains somewhat ambiguous. First, it is stated that the concept *dysfunctional* “inevitably involves normative judgments” (2). Later on, the concept of normativity is connected to the concepts of suffering and of clinical relevance, and its readability from “biological facts” or “biological measures” is denied (6). Finally, Walter defines *disorder*, following Graham, by being *harmful* and *undesirable for the subject* but adds the remark that these “normative criteria” are not dependent on the subject's appreciation (7)! Who, then, is the subject of these norms and valuations (appreciations), and where do their objectivity or legitimacy stem from? If one accepts the above solution regarding the alleged dichotomy of naturalism and normativism, this apparent aporia may be dissolved.

Marco Stier reports and discusses in his article a lot of arguments from current philosophical literature concerning the status of mental diseases. In my opinion, many of these arguments are in need of a critical examination. But I want to confine myself to an examination of Marco's first and main thesis: That diagnosis of mental disorder is dependent on the acceptance of socio-cultural norms and values. Slightly reformulated, his proof runs as follows:

1. Mental disorders are defined by the presence of *deviant behavior* (experience, emotion) and/or of *suffering*.
2. Deviance can only be recognized by comparing with (mental) *normality*.
3. Mental normality is defined by social and cultural norms.
4. The same holds true for the concepts of suffering and harm: What is recognized to be suffering or harm depends on socio-cultural norms and values.
5. Therefore, the diagnosis of mental disorder is dependent on the prevalence or acceptance of certain socio-cultural norms, and is varying relative to socio-cultural differences and changes.

Premise 1 is equivalent to the assumption that behavioral deviance and suffering are the decisive criteria of pathologicity in psychiatry. I am going to show that this premise is misguided in two respects, and leads to an inadequate view of biological psychiatry.

1. As soon as psychiatry succeeds in clarifying the etiopathogenesis of a mental disorder or disease regarding also its biological aspects, diagnostics of this disease no longer depends on behavioral or mental criteria: Once *pathognomonic somatic* markers or criteria of a disease are recognized, diagnosis of this disease may be secured or excluded by purely biological tests and procedures. To say more: If the disease in question is a disposition—a dispositional disease like “social phobia” or “tendency to panic attacks” –, then it is not even necessary that the patient at hand has shown the respective symptoms (behavior) at all, because diagnosis may be ascertained beforehand, e.g., by lab findings. The same shift from symptom-related diagnostics to biologically objectifiable methods is usual in somatic medicine, and is in accordance with the aforementioned principle that theoretical classification overwrites and overrides phenomenological criteria.
2. *Behavioral deviance* and *suffering* (or *harm*) are not genuine criteria of pathologicity. Already in the pre-scientific sense, there is no necessary or cogent connection between deviance and pathologicity. Instead, there may be many different causes and reasons of deviance, most of them without any relationship to disease. Additionally, deviance *may be*, but by no means is bound to be a *result* of some diseases, mental or somatic. Also, suffering and harm, as rather abstract categories, are not criteria of pathologicity, because these concepts are far too broad und undifferentiated. Rather, there are several definite, specific kinds of suffering that represent psychiatric criteria of pathologicity. To mention just a few examples:
  - the overwhelming, flooding kind of fear and angst that is typical for panic attacks
  - the “feeling of unfeelingness” in major depression
  - hallucinations, anhedonia (inability to experience pleasure), feelings of self-alienation and de-personalization, and catatonia in schizophrenics.

Symptoms of these kinds represent very specific forms of experience that, indeed, might be described as kinds of suffering, but are criteria of diseases and diagnosis only by their very special characteristics, not because they are cases of abstract suffering or harm. What is most important in the present context: These specific forms of experience and behavior are characterized by a very stable *cultural invariance* of their appearance and presentation; they do not vary relating to even very different cultural contexts. This is a well-ascertained insight of psychiatry, and it diametrally contradicts the thesis of Marco Stier.

## Footnotes

^1^The account of theoretical pathology in this section is a *very* condensed one. You will find far more elaborated versions in Hucklenbroich (2013, 2014).

^2^This controversy, meantime, lasts about several decades. For an overview and review see, e.g., Gottschalk-Mazouz (2008).
